# Thyroid markers and body composition predict LDL-cholesterol change in lean healthy women on a ketogenic diet: experimental support for the lipid energy model

**DOI:** 10.3389/fendo.2023.1326768

**Published:** 2023-12-21

**Authors:** Isabella D. Cooper, Claudio Sanchez-Pizarro, Nicholas G. Norwitz, David Feldman, Yvoni Kyriakidou, Kurtis Edwards, Lucy Petagine, Bradley T. Elliot, Adrian Soto-Mota

**Affiliations:** ^1^ Ageing Biology and Age-Related Diseases, School of Life Sciences, University of Westminster, London, United Kingdom; ^2^ Metabolic Diseases Research Unit, National Institute of Medical Science and Nutrition Salvador Zubiran, Mexico City, Mexico; ^3^ Tecnologico de Monterrey, Escuela de Medicina y Ciencias de la Salud, Mexico City, Mexico; ^4^ Harvard Medical School, Boston, MA, United States; ^5^ Citizen Science Foundation, Las Vegas, NV, United States

**Keywords:** cholesterol, ketogenic diet, lean mass hyper-responder, lipid energy model, cardiovascular risk

## Abstract

**Introduction:**

There is a large heterogeneity in LDL-cholesterol change among individuals adopting ketogenic diets. Interestingly, lean metabolically healthy individuals seem to be particularly susceptible, with an inverse association between body mass index and LDL-cholesterol change. The lipid energy model proposes that, in lean healthy individuals, carbohydrate restriction upregulates systemic lipid trafficking to meet energy demands. To test if anthropometric and energy metabolism markers predict LDL-cholesterol change during carbohydrate restriction.

**Methods:**

Ten lean, healthy, premenopausal women who habitually consumed a ketogenic diet for ≥6 months were engaged in a three-phase crossover study consisting of continued nutritional ketosis, suppression of ketosis with carbohydrate reintroduction, and return to nutritional ketosis. Each phase lasted 21 days. The predictive performance of all available relevant variables was evaluated with the linear mixed-effects models.

**Results:**

All body composition metrics, free T_3_ and total T_4_, were significantly associated with LDL-cholesterol change. In an interaction model with BMI and free T_3_, both markers were significant independent and interacting predictors of LDL-cholesterol change. Neither saturated fat, HOMA-IR, leptin, adiponectin, TSH, nor rT_3_ was associated with LDL-cholesterol changes.

**Discussion:**

Among lean, healthy women undergoing carbohydrate restriction, body composition and energy metabolism markers are major drivers of LDL-cholesterol change, not saturated fat, consistent with the lipid energy model.

## Graphical Abstract

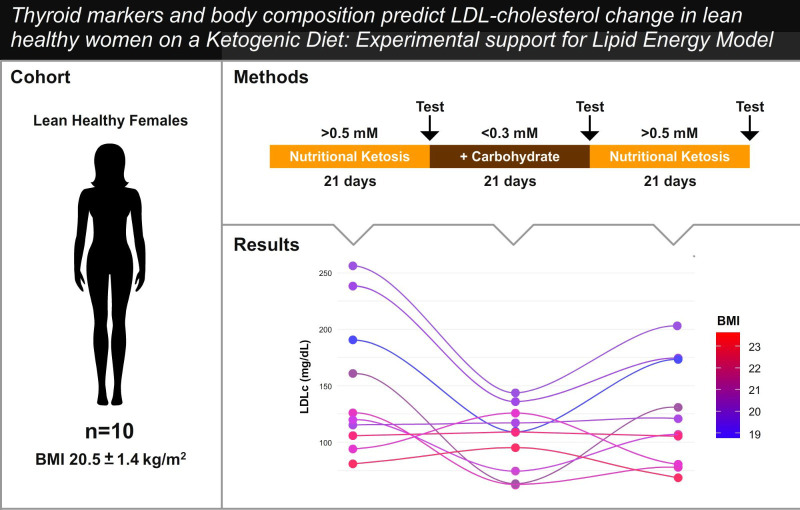

## 1 Introduction

Ketogenic diets (KDs) are becoming increasingly popular for the treatment of a wide range of chronic medical conditions, including epilepsy (1), neurodegenerative diseases (2), mental health disorders (3), diabetes (4), and many other chronic conditions not necessarily associated with an elevated body mass index (BMI). However, there is a large degree of heterogeneity in LDL-cholesterol (LDLc) changes to KD, with some individuals exhibiting decreases in LDLc, many exhibiting minimal change, and yet some exhibiting large increases on the order of 100s of mg/dL (5, 6).

At present, the sources of this heterogeneity are poorly understood and/or misunderstood, deterring the clinical implementation of this nutritional therapy. Interestingly (and counterintuitively), a major source of heterogeneity in the LDLc response may be BMI, with a normal BMI (≤~25 kg/m^2^) associated with larger increases in LDLc when compared with higher BMIs (6).

One hypothesis to explain this phenomenon is the lipid energy model (LEM) (7), which posits that the increase in LDLc exhibited by lean individuals on KD is a result of upregulation in systemic lipid trafficking to meet energy demands. Briefly, in lean individuals, a KD induces larger increases in circulating free fatty acids that are subsequently taken up by hepatocytes, resynthesized into triglycerides (TGs), and exported on very low-density lipoprotein (VLDL) particles. These VLDLs are depleted of their TG cargo by lipoprotein lipase (LPL) at peripheral adipocytes and oxidative tissues, resulting in TG-depleted LDL particles and an increase in HDL-cholesterol (HDLc) as HDL particles accept cholesterol from VLDL during LPL-mediated turnover.

The LEM also predicts that energy demand can influence LDLc changes too, as VLDL export and peripheral turnover should increase to meet the needs of increased peripheral tissue demands, all else being equal. In this three-phase crossover study, involving 10 lean, healthy women on KDs, we aim to confirm prior reports of an inverse association between BMI and LDLc on KD and test if thyroid hormones—well-documented regulators of energy metabolism—also predict LDLc change.

## 2 Methods

### 2.1 Ethical approval

Ethical approval was obtained by the College of Liberal Arts and Sciences Research Ethics Committee, University of Westminster, United Kingdom (ETH2122-0634). All procedures were conducted in accordance with the Declaration of Helsinki and UK legislation.

### 2.2 Study design

This study was an open-labeled, non-randomized crossover trial with three phases, each 21 days in duration: nutritional ketosis (NK; P1); suppressed ketosis (SuK) on higher carbohydrate UK Eatwell Guidelines, Standard United Kingdom (SUK; P2) guidelines; and returned to NK (P3). Participants were *n* = 10 healthy women. Recruitment criteria included healthy premenopausal lean (BMI < 25 kg/m^2^) women, who were not taking hormonal contraception, were non-smokers, and had been adherent to a KD for ≥6 months (mean 3.9 ± 2.3 years) to ensure sufficient time for metabolic adaptation. To confirm a state of NK, a daily capillary D-β-hydroxybutyrate (βHB) measurement was done between 16:00 and 18:00 before the evening meal during the 6 months prior to commencing the study. Standardized evening measurements were chosen as a more rigorous threshold (as compared with morning measurements after overnight fasting) to increase the probability that participants were in NK most of the time.

These analyses emerged from a larger recently published study. Further methodological details can be found here (8).

During the first 21-day phase, participants maintained NK, defined as βHB ≥0.5 mmol/L, and similar to those exhibited during their routine KD. On day 22, participants attended the Human Physiology Laboratory at the University of Westminster at 08:00 after an overnight fast (>12 h) for baseline anthropometric and biochemical testing.

Days 23–43 (21 days inclusive) marked phase 2 (SuK), in which participants aimed to suppress βHB to <0.3 mmol/L. To accomplish this, participants were instructed to adhere to the UK Eatwell Guideline (9), which recommends consuming a predominance of calories from carbohydrates (e.g., 55% kcal from carbohydrates on a 2,000 kcal diet is 275 g/day net carbohydrate), similar to most standard healthy eating guidelines worldwide. After the SuK phase on day 44, participants reported to the laboratory at 08:00 after an overnight fast for measurements, as previously. Days 45–65 marked phase 3, a return to NK.

All anthropometric measurements used Seca^®^ (mBCA 514 Medical Body Composition Analyzer, Gmbh&Co. KG, 996 Hamburg, Germany). Fat oxidation (FatOx) was extrapolated from the respiratory quotient (RQ) and measured with Quark RMR 1004 (COSMED srl, Rome, Italy). All participants lay supine for 15 min before measurements were taken.

Since thyroid hormones are well-documented regulators of energy expenditure (10, 11), we also sought to analyze if they are independent predictors of LDLc throughout the study. Dietary composition was assessed from daily dietary records on days 1, 14, and 21 of each phase using Food Processor^®^ (ESHA Research, Salem, Oregon, United States).

### 2.3 Statistical analysis

Data management and statistical analyses were performed using R version 2023.09.0 + 463, and all data manipulation was performed using *dplyr::*. To account for a repeated measures design, we used linear mixed-effects models with a random slope for each participant to compare the influence of different physiological variables on LDLc across the study. All models used the R function *lmerTest::lmer*.

Multidimensional predictive performance comparisons were made with *performance::compare_performance* and considered the Akaike information criterion (AIC), Bayes information criterion (BIC), root mean square error (RMSE), intraclass correlation coefficients (ICCs), and marginal *R* ^2^.

The sample size of the study was calculated based on pilot feasibility data with five participants who underwent all three phases with changes in insulin and insulin-like growth factor 1 (IGF-1) as the primary outcome. A sample size of *n* = 9 (*n* = 3 for IGF-1), was obtained using G*Power (v3.1) with an ICC = 0.5, an alpha level of 0.05, a target statistical power of 0.80, and a medium effect size of *f* = 0.5.

Our sensitivity analyses tested if other physiological variables, also known to influence lipid and lipoprotein metabolism [leptin, adiponectin, fasting insulin, homeostatic model for insulin resistance (HOMA-IR), fat oxidation, and saturated fat], took over the significance of BMI as an LDLc change predictor (suggestive of full mediation).

Additionally, we simulated data for every participant to test if a larger consumption of saturated fat (≥90% of total energy intake) would have changed our conclusions with the MS Excel function *randbetween* with lower and upper bounds (of 180 to 200 g/day for phases 1 and 3 and of 15 to 25 g/day for phase 2).

Finally, we tested if BMI and thyroid hormones were associated to rule out that their association with LDLc was simply due to potential collinearity.

The analysis code is available at https://github.com/AdrianSotoM/LMHRW.

## 3 Results

Participants had a mean BMI of 20.5 ± 1.4 kg/m^2^, were 32.3 ± 8.9 years of age, and had been on a KD for 3.9 ± 2.3 years. Adherence to each phase was confirmed with daily capillary βHB tests (NK, phase 1 = 1.9 ± 0.7; SuK, phase 2 = 0.1 ± 0.1; NK, phase 3 = 1.9 ± 0.6 mmol/L), and all 10 consistently achieved NK (βHB ≥ 0.5 mmol/L) and SuK (βHB < 0.03 mmol/L) as required by the study design. **Table 1** summarizes the changes in variables of interest throughout the study.

**Table 1:** Anthropometric and lipid metabolism measurements throughout the study.

	Phase 1		Phase 2		Phase 3		*p*-value
	Mean	(± SD)	Mean	(± SD)	Mean	(± SD)	
**BMI**	20.5	1.4	21.5	1.3	20.8	1.5	0.28
**Fat mass index**	5.5	1.0	6.1	1.0	5.7	1.0	0.37
**Fat-free mass index**	15.0	1.1	15.3	1.0	15.1	1.1	0.76
**Fat oxidation (%)**	97.8	4.2	87.8	13.6	98.7	1.9	0.01
**CHO oxidation (%)**	2.2	4.2	12.2	13.6	1.3	1.9	0.01
**Insulin (pmol/L)**	33.6	8.6	59.8	14.7	31.6	9.4	<0.001
**Glucose (mmol/L)**	4.4	0.5	5.1	0.6	4.4	0.3	0.003
**HOMA-IR**	1.1	0.3	2.3	0.7	1.0	0.4	<0.001
**Adiponectin (μg/mL)**	9.1	4.2	10.8	6.8	8.7	3.3	0.62
**LDLc (mg/dL)**	148.0	61.2	102.5	29.4	124.1	45.8	0.12
**HDLc (mg/dL)**	70.1	10.4	72.7	13.6	69.8	11.8	0.83
**Triglycerides (mg/dL)**	66.8	28.0	66.1	21.1	79.3	45.9	0.61
**fT_3_ (pmol/L)**	3.85	0.3	5.5	0.7	3.9	0.4	<0.001
**T_4_ (pmol/L)**	13.5	1.6	13.2	1.5	12.7	0.7	0.34
**TSH (mIU/L)**	1.4	0.7	1.6	0.8	1.3	0.8	0.67
**rT_3_ (nmol/L)**	0.3	0.1	0.3	0.1	0.3	0.1	0.64
**Resting metabolic rate (kcal/day)**	1,534	177	1,568	311	1,479	268	0.74
**Energy intake (kcal/day)**	1,834	236	1,830	232	1,757	176	0.66
**CHO intake (g/day)**	58.5	21.1	198.6	71.1	57.0	23.6	<0.001
**Protein (g/day)**	83.9	27.6	63.3	14.9	76.7	28.5	0.18
**Sat fat intake (g/day)**	33.7	12.4	20.9	7.1	28.9	15.1	0.07
**Simulated sat fat (g/day)**	196.3	3.3	19.9	3.3	194.6	3.3	<0.001

CHO, carbohydrate; sat fat, saturated fat.

As represented in **Table 2**, free triiodothyronine (fT_3_) and thyroxine (T_4_), but neither TSH nor reverse T_3_ (rT_3_), were associated with LDLc. All body composition markers—BMI, fat mass index (FMI), and fat-free mass index (FFMI), were associated with LDLc.

**Table 2:** Predictive performance of the models.

Model	Estimate	AIC	Marginal *R* ^2^	RMSE	*p*-value
**LDLc ~ fT_3_**	−20.4	312.2	11%	22.9	0.003
**LDLc ~ T_4_**	14.2	314.8	15%	29.6	0.03
**LDLc ~ TSH**	−5.6	320.1	0.07%	30.1	0.68
**LDLc ~ rT_3_**	−46.7	325.5	0.07%	30.1	0.59
**LDLc ~ BMI**	−25.1	307.8	42%	25.0	0.001
**LDLc ~ FMI**	−32.5	315.2	26%	24.3	0.01
**LDLc ~ FFMI**	−30.1	311.3	37%	29.0	0.01
**LDL ~ BMI: fT_3_**	BMI = −56.9 fT_3_ = −191.5 BMI:fT_3 =_ 8.5	305.6	38%	20.2	0.005 0.03 0.04

AIC, Akaike information criterion; marginal R^2^, variability explained by the fixed effects; RMSE, root mean square error.

**Figure 1:**
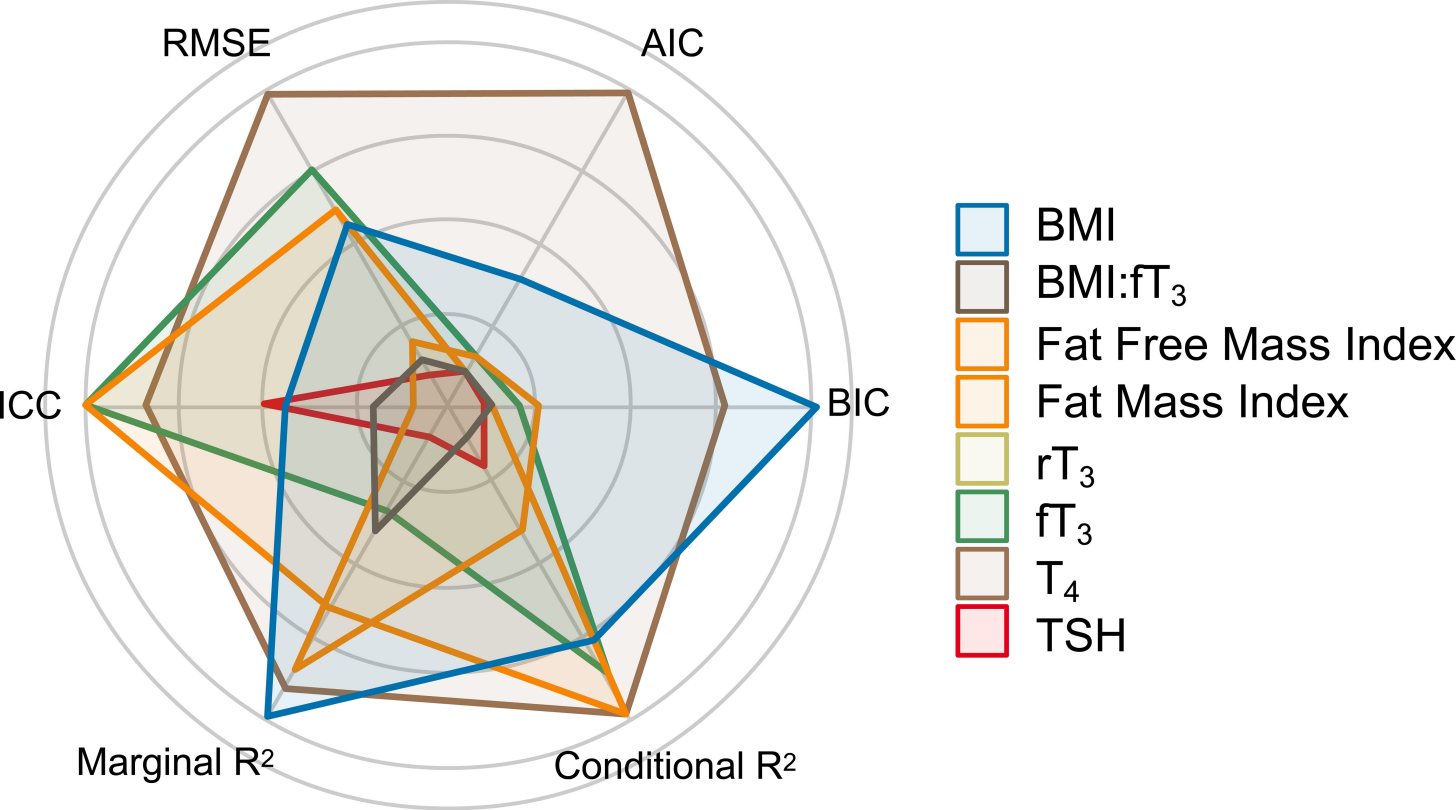
Multidimensional predictive performance comparison. AIC, Akaike information criterion; BIC, Bayes information criterion; conditional *R* ^2^, explained variability by both fixed and random effects; marginal *R* ^2^, explained variability by fixed effects; ICC, intraclass correlation coefficient; RMSE, root mean square error.

To build an interacting model between body composition and energy metabolism, fT_3_ and BMI were chosen due to their overall superior predictive performance. Both were independently and significantly interacting predictors of LDLc. **Figure 1** illustrates the multidimensional predictive performance comparisons in which the BMI:fT_3_ interaction model was the best predictor of all models (patent in the largest area of its polygon).

**Figure 2:**
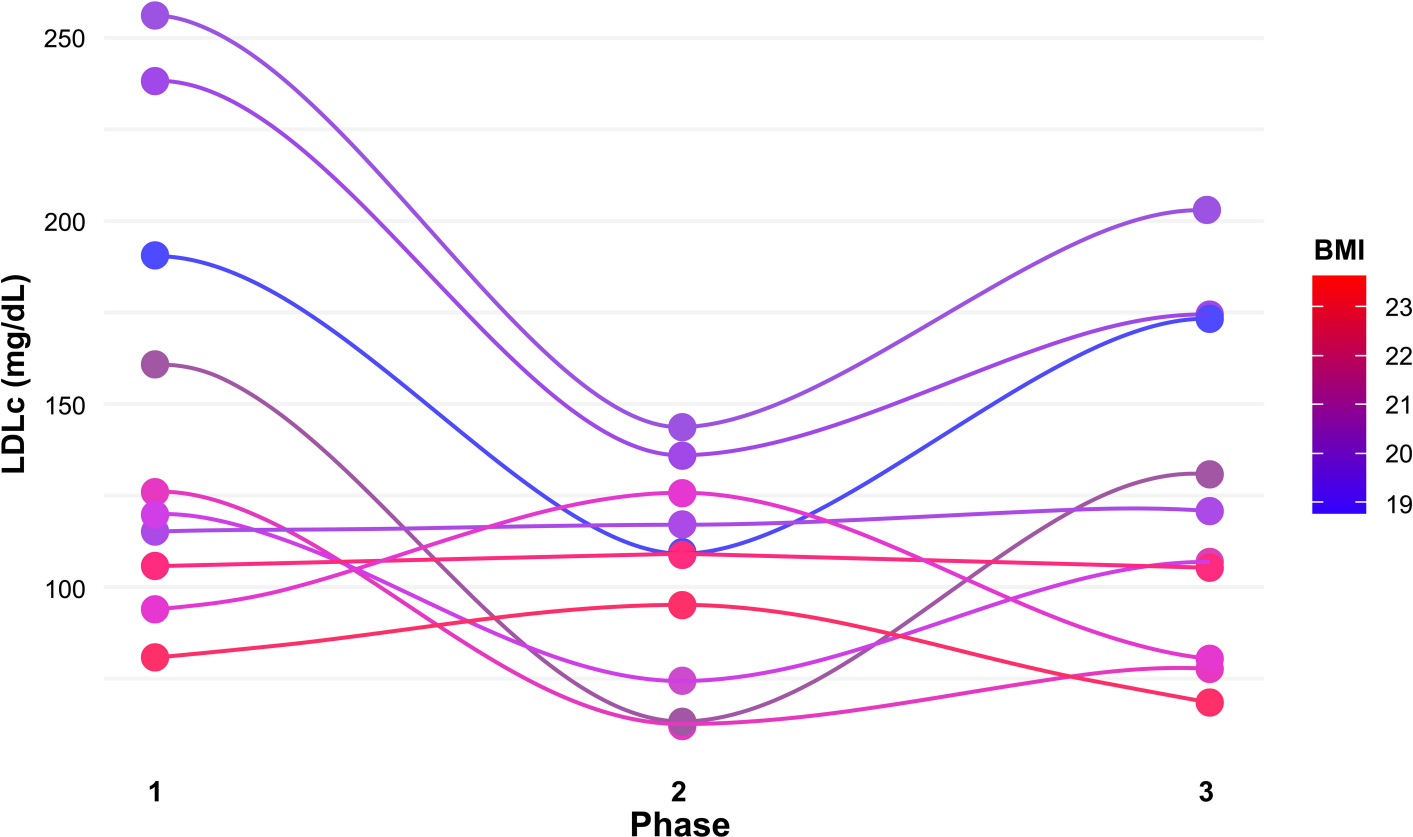
LDL-cholesterol change by phase and BMI.

As depicted in **Figure 2**, BMI remained stable throughout the study in all participants, and those with lower BMIs had higher LDLc during the first (NK) phase and larger LDLc changes across phases.

As shown in the sensitivity analyses in **Table 3**, BMI remained a robust and significant predictor of LDLc change despite the addition of any covariate, and no covariate remained significant after accounting for the effect of BMI. Even when tested against a saturated fat intake as high as 90% of total energy (1,800 kcal from pure saturated fat on a 2,000 kcal diet), BMI remained a significant, and virtually unchanged, predictor of LDLc change.

**Table 3:** Sensitivity analyses.

Model	Estimate	*p*-value
LDLc ~ BMI + adiponectin	BMI: −26.0 Adiponectin -1.9	0.001 0.26
LDLc ~ BMI + insulin	BMI: −22.1 Insulin: 0.4	0.02 0.40
LDLc ~ BMI + leptin	BMI: −33.7 Leptin: −0.2	0.01 0.84
LDLc ~ BMI + HOMA	BMI: −21.0 HOMA: −11.5	0.01 0.21
LDLc ~ BMI + ≥90% energy from saturated fat	BMI: −20.5 Sat fat: 0.09	0.02 0.23
fT_3_ ~ BMI	0.15	0.21
FT_4_ ~ BMI	−0.06	0.76
TSH ~ BMI	0.19	0.12
rT_3_ ~ BMI	0.003	0.84

Finally, BMI was not significantly associated with any of the measured thyroid markers.

## 4 Discussion

These experimental results are consistent with prior observational data in showing that BMI is inversely related to LDLc change on a KD (6). While prior data primarily report that lower BMI is associated with larger increases in LDLc upon adoption of KD, these data show the inverse is also true: those with lower BMI also exhibit larger decreases in LDLc upon carbohydrate reintroduction.

It is not surprising that all anthropometric measurements (BMI, FMI, FFMI) were significant predictors of LDLc given their expected collinearity among lean women. While BMI is an imperfect indicator of body composition, we focused on it as it is more widely available, and it yields the most conservative estimate size (*β* = −25) for our primary outcome and explains better the observed variability than the other anthropometric biomarkers (*R* ^2 =^ 0.42 vs. 0.26 and 0.37 for FMI and FFMI, respectively).

How do anthropomorphic measurements compare in predictive power to a more widely recognized influencer of LDLc levels: saturated fat intake? As shown in our sensitivity analysis, BMI dominates over saturated for determining LDLc change, even when ≥90% of total calories were derived from saturated fat.

Consistent with what has been documented in randomized crossover trials (12), fT_3_ was lower in the NK phases when compared with the SuK (higher carbohydrate) phase. Additionally, we found that fT_3_ and T_4_ (but not TSH or rT_3_) were predictive of LDLc change, which is consistent with the role of thyroid hormones in regulating systemic lipid energy trafficking to meet energy demands during carbohydrate restriction.

While this thyroid marker pattern may appear reminiscent of a “sick euthyroid syndrome,” a condition in which patients with critical illness demonstrate changes in peripheral thyroid hormone levels (13), we caution against this interpretation for the following reasons. i) We studied healthy participants. ii) As shown in **Table 1**, energy intake, weight, resting metabolic rate, TSH, and rT_3_ remained unchanged across throughout the study. iii) It is not yet clear how macronutrient shifts affect thyroid hormone sensitivity, and akin to what is observed in insulin or leptin resistance, the levels of a hormone may be inversely associated with its sensitivity.

A chief strength of this study included the rigor with which participants complied with the dietary regimen, as evidenced by all participants achieving consistent NK during the NK phases and consistent suppression of ketosis during the SuK phase, determined by blood capillary testing combined with dietary records. Additionally, all participants had a BMI ≤24 kg/m^2^ and, therefore, were “lean” and remained weight-stable, increasing the internal validity of this study.

Limitations include that our sample size was not calculated with LDLc changes as the primary outcome. However, it is unlikely that the negative results in our sensitivity analyses are due to low statistical power, as none had borderline statistical significance. Another limitation is the relatively short duration of each phase, as noticeable from the fact that 21 days was not sufficient for participants to return to baseline (elevated) LDLc on a KD. Of note, it has been reported that some physiological adaptations to macronutrient shifting may persist after 3 months (14). Nonetheless, since all participants followed a KD for ≥6 months before enrolment, it is likely that a longer follow-up would only have caused phase 3 to mirror phase 1 more closely and have strengthened the main findings in this work. Future studies will investigate the potential role of other potentially relevant hormonal modulators in the LEM.

### 4.1 Conclusions

The amplitude of LDLc changes (both increases with KD adoption and decreases with carbohydrate reintroduction) is larger when BMI is lower. Additionally, fT_3_ is an independent predictor of LDLc changes, separate from BMI. These findings are consistent with and build upon the lipid energy model and provide useful insight for lean people considering adopting carbohydrate-restricted ketogenic diets.

## Data availability statement

The original contributions presented in the study are included in the article/supplementary material. Further inquiries can be directed to the corresponding author.

## Ethics statement

The studies involving humans were approved by the College of Liberal Arts and Sciences Research Ethics Committee, University of Westminster, United Kingdom (ETH2122-0634). The studies were conducted in accordance with the local legislation and institutional requirements. The participants provided their written informed consent to participate in this study.

## Author contributions

IC: Conceptualization, Funding acquisition, Investigation, Methodology, Project administration, Resources, Supervision, Validation, Writing – original draft, Writing – review & editing. CS-P: Data curation, Formal analysis, Investigation, Methodology, Writing – original draft, Writing – review & editing. NN: Conceptualization, Formal analysis, Investigation, Methodology, Supervision, Visualization, Writing – original draft, Writing – review & editing. DF: Conceptualization, Investigation, Methodology, Supervision, Visualization, Writing – review & editing. YK: Investigation, Methodology, Project administration, Writing – review & editing. KE: Investigation, Methodology, Project administration, Writing – review & editing. LP: Investigation, Methodology, Project administration, Writing – review & editing. BE: Conceptualization, Project administration, Supervision, Writing – review & editing. AS-M: Conceptualization, Data curation, Formal analysis, Investigation, Methodology, Supervision, Visualization, Writing – original draft, Writing – review & editing.
